# Supplementary material for: Modeling glioblastoma heterogeneity as a dynamic network of cell states
Source: Mol Syst Biol. 2021 Sep 16;17(9):e10105. doi: 10.15252/msb.202010105 (PMC8444284; doi:10.15252/msb.202010105)
Supplement: Supplementary file 6 — Source Data for Figure 5 [file MSB-17-e10105-s004.zip › Figure5A_sourcedata/GSEA_3071/hallmarks_stateI.GseaPreranked.1623742531165/HALLMARK_MYC_TARGETS_V1.html]

Details for gene set HALLMARK\_MYC\_TARGETS\_V1[GSEA]

|  || Dataset | state13071 |
| Phenotype | NoPhenotypeAvailable |
| Upregulated in class | na\_neg |
| GeneSet | HALLMARK\_MYC\_TARGETS\_V1 |
| Enrichment Score (ES) | -0.44539502 |
| Normalized Enrichment Score (NES) | -2.2676506 |
| Nominal p-value | 0.0 |
| FDR q-value | 4.0613025E-4 |
| FWER p-Value | 0.002 |
Table: GSEA Results Summary

  

Fig 1: Enrichment plot: HALLMARK\_MYC\_TARGETS\_V1      
 Profile of the Running ES Score & Positions of GeneSet Members on the Rank Ordered List

  

| PROBE | GENE SYMBOL | GENE\_TITLE | RANK IN GENE LIST | RANK METRIC SCORE | RUNNING ES | CORE ENRICHMENT || 1 | XPOT |  |  | 314 | 0.658 | -0.0761 | No |
| 2 | PRDX4 |  |  | 494 | 0.519 | -0.1162 | No |
| 3 | EIF2S2 |  |  | 1329 | 0.263 | -0.3535 | No |
| 4 | CTPS1 |  |  | 1540 | -0.261 | -0.4087 | No |
| 5 | RPLP0 |  |  | 1551 | -0.262 | -0.4054 | No |
| 6 | NDUFAB1 |  |  | 1635 | -0.272 | -0.4232 | No |
| 7 | RSL1D1 |  |  | 1675 | -0.277 | -0.4280 | No |
| 8 | BUB3 |  |  | 1687 | -0.278 | -0.4247 | No |
| 9 | VDAC3 |  |  | 1728 | -0.284 | -0.4297 | No |
| 10 | PSMD8 |  |  | 1730 | -0.284 | -0.4233 | No |
| 11 | TRIM28 |  |  | 1789 | -0.292 | -0.4333 | No |
| 12 | HNRNPA2B1 |  |  | 1819 | -0.296 | -0.4348 | No |
| 13 | POLD2 |  |  | 1820 | -0.296 | -0.4278 | No |
| 14 | RUVBL2 |  |  | 1829 | -0.297 | -0.4231 | No |
| 15 | EIF4E |  |  | 1900 | -0.305 | -0.4363 | No |
| 16 | RAN |  |  | 1907 | -0.306 | -0.4309 | No |
| 17 | KPNA2 |  |  | 1944 | -0.312 | -0.4340 | No |
| 18 | SNRPA1 |  |  | 1984 | -0.316 | -0.4379 | Yes |
| 19 | TCP1 |  |  | 1990 | -0.316 | -0.4319 | Yes |
| 20 | VBP1 |  |  | 1993 | -0.317 | -0.4250 | Yes |
| 21 | EIF4A1 |  |  | 2002 | -0.317 | -0.4198 | Yes |
| 22 | SNRPD2 |  |  | 2024 | -0.321 | -0.4184 | Yes |
| 23 | EIF1AX |  |  | 2103 | -0.335 | -0.4332 | Yes |
| 24 | PHB2 |  |  | 2129 | -0.339 | -0.4325 | Yes |
| 25 | MRPL23 |  |  | 2130 | -0.339 | -0.4245 | Yes |
| 26 | CCT2 |  |  | 2163 | -0.345 | -0.4257 | Yes |
| 27 | GSPT1 |  |  | 2177 | -0.348 | -0.4213 | Yes |
| 28 | NCBP2 |  |  | 2204 | -0.353 | -0.4205 | Yes |
| 29 | CYC1 |  |  | 2208 | -0.354 | -0.4130 | Yes |
| 30 | UBE2E1 |  |  | 2281 | -0.369 | -0.4254 | Yes |
| 31 | GLO1 |  |  | 2292 | -0.371 | -0.4195 | Yes |
| 32 | YWHAQ |  |  | 2304 | -0.373 | -0.4139 | Yes |
| 33 | NOP56 |  |  | 2333 | -0.380 | -0.4131 | Yes |
| 34 | GNL3 |  |  | 2342 | -0.382 | -0.4064 | Yes |
| 35 | MCM2 |  |  | 2349 | -0.383 | -0.3991 | Yes |
| 36 | RRP9 |  |  | 2373 | -0.388 | -0.3966 | Yes |
| 37 | HSPD1 |  |  | 2396 | -0.392 | -0.3938 | Yes |
| 38 | IMPDH2 |  |  | 2417 | -0.396 | -0.3903 | Yes |
| 39 | HNRNPU |  |  | 2434 | -0.398 | -0.3855 | Yes |
| 40 | YWHAE |  |  | 2453 | -0.402 | -0.3813 | Yes |
| 41 | HPRT1 |  |  | 2454 | -0.402 | -0.3718 | Yes |
| 42 | HNRNPA1 |  |  | 2455 | -0.402 | -0.3623 | Yes |
| 43 | SNRPG |  |  | 2459 | -0.404 | -0.3537 | Yes |
| 44 | C1QBP |  |  | 2482 | -0.407 | -0.3504 | Yes |
| 45 | SNRPA |  |  | 2515 | -0.417 | -0.3499 | Yes |
| 46 | UBA2 |  |  | 2517 | -0.418 | -0.3403 | Yes |
| 47 | CDC20 |  |  | 2540 | -0.425 | -0.3367 | Yes |
| 48 | TXNL4A |  |  | 2558 | -0.428 | -0.3316 | Yes |
| 49 | SRM |  |  | 2568 | -0.432 | -0.3240 | Yes |
| 50 | POLE3 |  |  | 2570 | -0.432 | -0.3141 | Yes |
| 51 | RPS2 |  |  | 2601 | -0.439 | -0.3124 | Yes |
| 52 | SRSF7 |  |  | 2619 | -0.445 | -0.3069 | Yes |
| 53 | SRSF3 |  |  | 2631 | -0.447 | -0.2995 | Yes |
| 54 | HNRNPA3 |  |  | 2634 | -0.449 | -0.2895 | Yes |
| 55 | EIF2S1 |  |  | 2638 | -0.450 | -0.2798 | Yes |
| 56 | DUT |  |  | 2640 | -0.450 | -0.2694 | Yes |
| 57 | RNPS1 |  |  | 2641 | -0.450 | -0.2588 | Yes |
| 58 | SET |  |  | 2652 | -0.455 | -0.2509 | Yes |
| 59 | CCNA2 |  |  | 2667 | -0.462 | -0.2441 | Yes |
| 60 | NME1 |  |  | 2669 | -0.463 | -0.2335 | Yes |
| 61 | NHP2 |  |  | 2684 | -0.468 | -0.2265 | Yes |
| 62 | LSM7 |  |  | 2690 | -0.470 | -0.2168 | Yes |
| 63 | MCM6 |  |  | 2699 | -0.471 | -0.2080 | Yes |
| 64 | SNRPD1 |  |  | 2701 | -0.471 | -0.1972 | Yes |
| 65 | RPL14 |  |  | 2779 | -0.497 | -0.2079 | Yes |
| 66 | SRSF2 |  |  | 2782 | -0.497 | -0.1968 | Yes |
| 67 | FBL |  |  | 2804 | -0.502 | -0.1910 | Yes |
| 68 | CDK2 |  |  | 2863 | -0.526 | -0.1955 | Yes |
| 69 | PPM1G |  |  | 2907 | -0.545 | -0.1952 | Yes |
| 70 | TRA2B |  |  | 2933 | -0.563 | -0.1892 | Yes |
| 71 | SMARCC1 |  |  | 2973 | -0.584 | -0.1868 | Yes |
| 72 | TFDP1 |  |  | 3027 | -0.617 | -0.1877 | Yes |
| 73 | CDC45 |  |  | 3081 | -0.661 | -0.1875 | Yes |
| 74 | MAD2L1 |  |  | 3099 | -0.675 | -0.1765 | Yes |
| 75 | CNBP |  |  | 3113 | -0.686 | -0.1641 | Yes |
| 76 | PA2G4 |  |  | 3116 | -0.690 | -0.1484 | Yes |
| 77 | RFC4 |  |  | 3129 | -0.701 | -0.1353 | Yes |
| 78 | MCM5 |  |  | 3132 | -0.706 | -0.1192 | Yes |
| 79 | HNRNPD |  |  | 3157 | -0.738 | -0.1088 | Yes |
| 80 | USP1 |  |  | 3183 | -0.762 | -0.0981 | Yes |
| 81 | RRM1 |  |  | 3215 | -0.801 | -0.0882 | Yes |
| 82 | ODC1 |  |  | 3257 | -0.860 | -0.0799 | Yes |
| 83 | RANBP1 |  |  | 3290 | -0.911 | -0.0677 | Yes |
| 84 | PCNA |  |  | 3319 | -0.963 | -0.0531 | Yes |
| 85 | MCM4 |  |  | 3341 | -1.006 | -0.0354 | Yes |
| 86 | DEK |  |  | 3344 | -1.025 | -0.0118 | Yes |
| 87 | MCM7 |  |  | 3384 | -1.147 | 0.0039 | Yes |
| 88 | TYMS |  |  | 3436 | -1.391 | 0.0219 | Yes |
Table: GSEA details [plain text format]

  

Fig 2: HALLMARK\_MYC\_TARGETS\_V1: Random ES distribution      
 Gene set null distribution of ES for **HALLMARK\_MYC\_TARGETS\_V1**
